# Using Artificial Intelligence to Stratify Normal versus Abnormal Chest X-rays: External Validation of a Deep Learning Algorithm at East Kent Hospitals University NHS Foundation Trust

**DOI:** 10.3390/diagnostics13223408

**Published:** 2023-11-09

**Authors:** Sarah R. Blake, Neelanjan Das, Manoj Tadepalli, Bhargava Reddy, Anshul Singh, Rohitashva Agrawal, Subhankar Chattoraj, Dhruv Shah, Preetham Putha

**Affiliations:** 1 East Kent Hospitals University NHS Foundation Trust, Ashford TN24 OLZ, UK; s.blake4@nhs.net; 2 Qure.ai, Mumbai 400063, Maharashtra, India; manoj.tadepalli@qure.ai (M.T.); bhargava.reddy@qure.ai (B.R.); anshul.singh@qure.ai (A.S.); rohit.agrawal@qure.ai (R.A.); dhruv.shah@qure.ai (D.S.); preetham.putha@qure.ai (P.P.)

**Keywords:** computer-aided detection, deep learning, CXR, artificial intelligence, NHS Foundation Trust, qXR

## Abstract

*Background:* The chest radiograph (CXR) is the most frequently performed radiological examination worldwide. The increasing volume of CXRs performed in hospitals causes reporting backlogs and increased waiting times for patients, potentially compromising timely clinical intervention and patient safety. Implementing computer-aided detection (CAD) artificial intelligence (AI) algorithms capable of accurate and rapid CXR reporting could help address such limitations. A novel use for AI reporting is the classification of CXRs as ‘abnormal’ or ‘normal’. This classification could help optimize resource allocation and aid radiologists in managing their time efficiently. *Methods:* qXR is a CE-marked computer-aided detection (CAD) software trained on over 4.4 million CXRs. In this retrospective cross-sectional pre-deployment study, we evaluated the performance of qXR in stratifying normal and abnormal CXRs. We analyzed 1040 CXRs from various referral sources, including general practices (GP), Accident and Emergency (A&E) departments, and inpatient (IP) and outpatient (OP) settings at East Kent Hospitals University NHS Foundation Trust. The ground truth for the CXRs was established by assessing the agreement between two senior radiologists. *Results:* The CAD software had a sensitivity of 99.7% and a specificity of 67.4%. The sub-group analysis showed no statistically significant difference in performance across healthcare settings, age, gender, and X-ray manufacturer. *Conclusions:* The study showed that qXR can accurately stratify CXRs as normal versus abnormal, potentially reducing reporting backlogs and resulting in early patient intervention, which may result in better patient outcomes.

## 1. Introduction

The chest radiograph (CXR) is the most frequently performed radiological examination worldwide, with an average of 238 erect-view chest X-ray images acquired per 1000 people annually in developing countries [1]. In the United States alone, approximately 129 million CXR images were estimated to have been acquired in 2006 [2]. The widespread demand and availability of CXRs can be attributed to their cost-effectiveness, low radiation-dose exposure, and reasonable sensitivity in detecting medical conditions. As the initial imaging modality, the CXR remains a central component in screening, diagnosing, and managing different health conditions [3].

Due to the rising demand for health services, an escalating backlog of chest X-rays has emerged as a critical concern [4]. This growing backlog has cascading effects on patient care. Delayed reporting may translate into longer patient wait times in the initiation of management, an issue that can cause significant clinical and operational consequences. Any delay in initiating the timely management of pathology can have a negative impact on patient outcomes. This becomes more critical in acute settings, where delayed diagnosis could lead to permanent disability or death [5]. Extended wait times can strain hospital resources, leading to longer hospital stays, increased costs, and worse patient outcomes [6]. While a significant proportion of CXRs might be classified as ‘normal’ or without significant findings, the challenge for radiologists is ensuring that no abnormalities present on CXRs are missed while reporting. Therefore, every CXR, regardless of perceived urgency, requires thorough evaluation. Given the volume of ‘normal’ CXRs, radiologists spend a significant portion of their time evaluating CXRs where no pathology is present. If these ‘normal’ CXRs could be swiftly and accurately segregated, radiologists could focus their expertise and time on more complex cases, potentially alleviating some of the backlog and expediting patient care. An AI system, specifically trained to segregate normal CXRs with a very low false-negative rate, could serve as a robust triage tool, aiding radiologists in streamlining their workflow. Over the past few years, there have been several advances in the application of deep learning to the interpretation of medical images [7,8,9], presenting an opportunity to address this challenge.

Deep learning systems for analyzing chest X-rays have been developed for automating the detection of radiological signs of tuberculosis [10,11,12], pneumonia [13], COVID-19 [14,15], pneumothorax, and lung nodules [16,17]. The WHO has recently endorsed computer-aided detection (CAD) technologies for tuberculosis (TB) diagnosis in individuals aged 15 and above as a replacement for human interpreters in the assessment of digital chest radiographs for TB screening and triage [18]. This recommendation stems from a growing body of research affirming the effectiveness and safety of CAD systems in TB screening across diverse geographical regions. This underscores the potential for the clinical implementation of AI systems in an automated or semi-automated capacity. Nonetheless, the performance of AI-based computer-aided detection (CAD) systems may exhibit variability when applied in novel clinical contexts [19]. Therefore, it is of paramount importance to rigorously assess AI systems using datasets that accurately represent the local population to ascertain whether their advertised performance aligns with the specific characteristics of the local population. This verification process is essential before considering deployment in active clinical settings, as patient safety is the foremost concern.

In this pre-deployment study conducted at East Kent Hospitals University NHS Foundation Trust, we aim to evaluate the performance and clinical utility of a fully automated computer-aided detection (CAD) system, qXR 4.0 (CE MDR Class IIb, Qure.ai), in stratifying chest X-rays (CXRs) as normal or abnormal, with a focus on its effectiveness in various healthcare settings and demographic groups, as well as its potential impact on optimizing the workload of reporting radiologists while ensuring patient safety. Multiple clinical applications of qXR have undergone evaluation in prior studies, studying its generalizability across various healthcare settings and geographic locations. Globally, qXR has been employed in diagnostic pathways for tuberculosis (TB) [10,11,12,20,21], lung nodules [13,16], and COVID-19 [14], as well as in the classification of CXRs as normal or abnormal [22,23,24]. A study by Govindarajan et al. [22] found that qXR improved the diagnostic accuracy and turnaround time for reporting abnormal CXRs in a prospective setting. These publications indicate the potential utility of AI software such as qXR 4.0 (CE MDR Class IIb, Qure.ai) in enhancing diagnostic precision and expediting reporting times for CXRs.

## 2. Materials and Methods

### 2.1. Device Description

The AI algorithms behind qXR are convolutional neural networks (CNN) trained on a dataset of 4.4 million CXRs and radiology reports collected from healthcare centers across the globe. Originally, the ground truth for training the algorithms was a combination of natural language processing (NLP)-generated labels and pixel-level annotations by board-certified radiologists. When run on a CXR, the software produces an abnormality score—a real-valued confidence score between 0 and 1—indicating the presence of abnormal findings. Additionally, for CXRs with abnormality scores above a pre-determined threshold, qXR produces a list of confidence scores and localization contours, indicating the presence and location of various abnormal findings, including but not limited to opacities, consolidations, pleural effusions, fibrosis, cardiomegaly, atelectasis, nodules, pneumothoraces, blunted costophrenic (CP) angles, raised diaphragms, tracheal shifts, cavities, rib fractures, Hilar lymphadenopathy, scoliosis, emphysema, and pneumoperitoneum. A detailed device description is provided in Appendix A.

### 2.2. Dataset

We retrospectively collected 1040 CXRs from East Kent Hospitals University NHS Foundation Trust. Data were sourced from multiple clinical settings, including general practices (GP), Accident and Emergency (A&E) departments, and inpatient (IP) and outpatient (OP) settings. The CXRs were filtered at the source according to the following inclusion and exclusion criteria:

- Inclusion Criteria:
  – Age ≥18 years.
  – PA/AP view.
  – Minimum image resolution of 1440 × 1440.
  – Minimum of 10 gray levels.
- Exclusion Criteria:
  – Lateral CXRs.
  – Incomplete view of the chest.
  – CXR images containing excessive motion artifacts.

As a result, only PA- or AP-view CXRs of adult patients (18 years and above) were included. Additionally, CXRs with motion artifacts, inadequate coverage of anatomy, gross patient rotation, and external metal or clothing artifacts were excluded from the study. The eligible CXRs were processed using qXR v4.0. The results were generated in JSON format and secondary capture DICOM (Digital Imaging and Communications in Medicine) files, where localization contours of abnormal findings were burned as an overlay onto the original images. All data management and processing was performed on a server with qXR v4.0 installed in the East Kent Hospitals University NHS Foundation Trust in compliance with the regulations specified by the local information governance and information technology team.

### 2.3. Establishing the Ground Truth

The ground truth for the CXRs sourced for this pre-deployment evaluation was determined through a concordance evaluation between two senior board-certified radiologists (M.D. Radiodiagnosis) with 10 and 12 years of experience. In scenarios where the radiologists rendered discordant interpretations, the case was categorized as abnormal in the final ground truth if either radiologist labeled the CXR as abnormal. This protocol ensured that a CXR indicating any potential abnormal finding would be deemed abnormal for the purpose of this evaluation, maximizing safety against false negatives. Conversely, a case was only labeled as normal in the final ground truth when both radiologists unanimously labeled the case as normal.

### 2.4. Statistical Analysis

The primary objective of the evaluation was to assess the performance of qXR in stratifying normal CXRs while minimizing false negatives to enable deployment in a live scenario. A false-negative result is considered a worse outcome than a false-positive one in preliminary diagnostic investigations. Hence, we chose NPV to calculate the sample sizes for this study [24]. It was estimated that a minimum of 800 CXRs, i.e., 400 abnormal and 400 normal CXRs, would be required for this study. At an expected NPV of 85%, the estimated sample size would provide a power of 80% with 5% precision [25,26]. We assessed qXR’s performance by comparing its predictions to the ground truth, derived as described in Section 2.3. We report the sensitivity, specificity, NPV, and PPV values derived by thresholding the continuous-valued outputs using the default device threshold provided by the manufacturer. Sub-group analyses were conducted based on healthcare setting, age, gender, and X-ray machine manufacturers to assess the robustness and generalizability of qXR’s performance across these parameters. The Wilson score interval was employed to calculate the 95% confidence intervals (CI) for sensitivity, specificity, NPV, and PPV [26]. All statistical analyses were conducted using Python version 3.9.7, scikit-learn 0.20, and pandas 3.8.

## 3. Results

**Figure 1:**
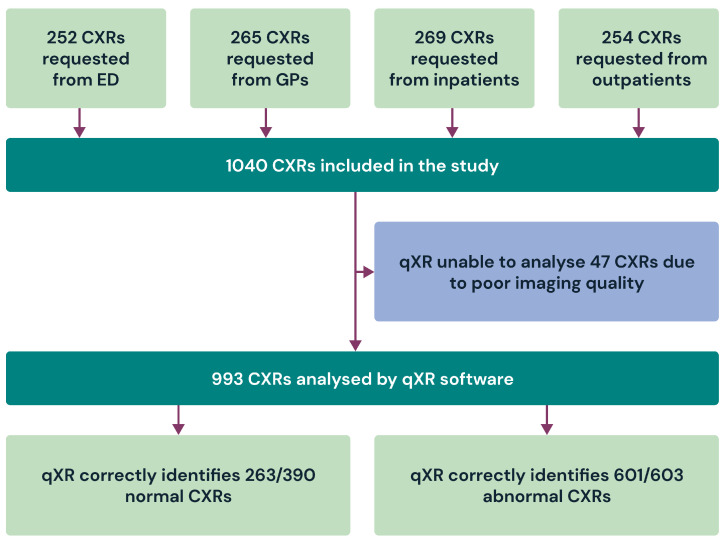
A total of 1040 CXRs were sourced from A&Es (252), GPs ( 265), and inpatient (269) and outpatient (254) settings. Among these, 47 CXRs were excluded due to poor image quality. Results are reported on 993 CXRs. qXR correctly identified 263 out of 390 normal CXRs and 601 out of 603 abnormal CXRs.

The initial dataset consisted of 1040 chest X-rays (CXRs) collected from various clinical settings. After excluding 47 CXRs due to the inclusion and exclusion criteria of the device, a total of 993 CXRs were included in the study analysis. Two radiologists independently provided ground-truth reports for all 993 CXRs. In addition to categorizing each CXR as either ‘normal’ or ‘abnormal’, the radiologists evaluated abnormalities individually, marking ‘yes’ or ‘no’ for the presence or absence of opacities, pneumothoraces, pleural effusions, cardiomegaly, Hilar enlargements, and nodules. As explained in Section 2.3, a CXR was classified as ‘normal’ only when both radiologists unanimously agreed on the ‘normal’ label. Similarly, the absence of an individual abnormality was established only when both radiologists unanimously confirmed the absence of an abnormality. This study is summarized in Figure 1 as a flow diagram.

We used the following definitions for reporting the performance of qXR: ‘Opacity’ indicates any abnormal radio-opacity, including consolidations, fibrosis, nodules, masses, calcifications, and edema; ‘Pleural Effusion’ includes cases with blunting of the costophrenic angle and effusions; and ‘Any Abnormality’ includes opacities, pneumothoraces, pleural effusions, Hilar enlargements, blunted costophrenic angles, cardiomegaly, and cavities. Out of the 993 eligible CXRs, 390 (39.3%) were categorized as ‘normal’ according to the ground truth because both radiologists unanimously labeled them as normal. The remaining 603 (60.7%) CXRs were categorized as ‘abnormal’ because at least one radiologist labeled them as abnormal. Discordant interpretations between the radiologists were observed in 167 CXRs (16.8%), a rate that is consistent with the inter-reader variability reported in the existing literature [27,28]. According to the ground-truth protocol, all cases with discordant interpretations were categorized as ‘abnormal’ for the purposes of this evaluation.

The baseline data characteristics are reported in Table 1. The distribution of eligible CXRs from different clinical settings—general practices (GP), Accident and Emergency (A&E) departments, and outpatient (OP) and inpatient (IP) settings—was approximately equal, with each setting contributing approximately 25% of the total CXRs. IP setting had the highest prevalence of abnormal CXRs (194/255), whereas the GP setting had the lowest prevalence of abnormal CXRs (109/255), aligning with real-world expectations. The age of the assessed study population ranged from 18 to 104 years, with a mean age of 65.05. Female patients accounted for 49.7% of the CXRs included in the study. There was representation from three X-ray manufacturers—Philips, Fujifilm, and Kodak. More than 50% of the X-rays included in the study were from Philips X-ray systems. Additionally, the final study dataset included 358 AP-view CXRs and 635 PA-view CXRs. The detailed performance evaluation stratified by target abnormalities is given in Table 2.

The CAD software demonstrated high accuracy in classifying abnormal CXRs, correctly identifying 601 out of a total of 603 scans. Additionally, the software accurately stratified 67.4% (263 out of 390) of normal CXRs but failed to detect abnormal findings in two instances. These results indicate a substantial opportunity for reducing the workload associated with reporting normal cases without compromising patient safety. CXRs featuring implants, medical devices, or age-related non-specific changes were designated as abnormal by qXR, ensuring additional scrutiny by radiologists in a real-world setting. We report the sensitivity, specificity, and diagnostic predictive values in Table 1.

The sub-group analysis of the results, stratified by healthcare settings, age, gender, and manufacturer, is detailed in Table 3 and Table 4. Notably, there was no statistically significant variance in the sensitivity and specificity values observed across all sub-groups, underscoring the software’s robustness in these parameters. The negative predictive value in stratifying normal vs. abnormal CXRs was consistent across all referral sources. Furthermore, the algorithm’s performance exhibited uniformity across age, gender, and manufacturer sub-groups.

## 4. Discussion

Given the escalating global demand for healthcare services and the ubiquitous role of chest X-rays as the most commonly utilized radiological examination, the strain on radiology services is growing rapidly. A radiology workforce census report by the Royal College of Radiologists (RCR) in 2022 found a workforce shortfall of 30%, which is projected to increase to 40% by 2027 [29]. This rising demand, coupled with a chronic shortage of radiologists, leads to significant backlogs in reporting. The delayed interpretation of these essential diagnostic tests can impede timely clinical management, potentially adversely affecting patient outcomes. It is vital to identify avenues for efficient yet reliable triage methods to streamline the reporting process.

However, the deployment of AI in healthcare necessitates a thorough understanding of the potential variability in AI performance dictated by local data characteristics—an issue termed ’the generalisation gap’. This consideration becomes acutely important in healthcare, where ensuring patient safety is the foremost concern. The regional prevalence of specific diseases, which demographically influences the presentation of findings and leads to variations in imaging protocols, can potentially exert considerable influence on the performance of AI tools. Consequently, local validation of AI systems is critical to ensure their effectiveness and safe application in different healthcare contexts. One effective approach is to initiate pre-deployment exercises, where the system is evaluated using data that accurately represent the local patient populace. This not only ensures a higher degree of precision but also fosters broader applicability, facilitating smooth transitions into larger pilot studies. Furthermore, pre-deployment assessments offer a valuable opportunity to identify and rectify potential biases within the AI system. By closely examining the system’s performance on locally representative datasets, any inherent biases can be identified and addressed. It is worth noting that many AI systems output continuous variables, and a critical aspect of their application is establishing a threshold to make binary decisions. This threshold determination can influence the system’s diagnostic accuracy. A guidance document by the WHO in 2021 recommends locally calibrating the thresholds to support the effective use of CAD for TB screening [30]. Pre-deployment exercises provide an ideal platform to locally calibrate these thresholds, fine-tuning the CAD system for optimal performance in clinical workflows. On the other hand, regulatory bodies, recognizing the vital role of continuous monitoring, sometimes mandate post-market surveillance. This involves the ongoing assessment of the tool’s performance post-deployment to quickly identify and address any issues that may not have been evident during the initial evaluations. In the ever-evolving landscape of healthcare delivery, it is essential for healthcare and software providers to establish standardized validation frameworks [31] and monitoring systems to leverage the potential of AI while upholding the highest standards of patient care.

Our retrospective cross-sectional pre-deployment evaluation provides evidence for the clinical utility of CAD software as an effective triage tool, paving the way for a larger prospective pilot in a live setting. The software achieved a sensitivity of 99.7% for abnormal CXRs and correctly stratified 67.4% of normal CXRs. These results were consistently observed across diverse clinical settings. Our sub-group analysis found that the performance of CAD was consistent across age, gender, and machine manufacturers. The potential impact of AI-based solutions like qXR becomes more salient in the context of existing radiologist shortages. By enabling high-confidence differentiation of ‘normal’ and ‘abnormal’ CXRs, the software could allow reporting radiologists to optimize their workflows. The low false-negative rate implies that the software can be reliably deployed in clinical settings, playing the role of a cautious, overcalling junior radiologist by flagging any CXRs that warrant further review. Additionally, by correctly identifying almost 70% of normal CXRs, the software demonstrates the potential for significant workload reduction. In a real-world clinical environment burdened by backlogs, such efficiencies could pave the way for faster diagnoses and streamlined patient management without compromising patient safety.

## 5. Limitations

Although this study generated valuable evidence, it has certain inherent limitations that must be acknowledged. This study relies on retrospective data. AI’s longitudinal impact on clinical decisions was not assessed. Although the overall normal and abnormal samples were sufficiently powered, the individual abnormalities were not independently powered for the study due to the aim of the project and the logistical constraints. The lack of histopathological or clinical diagnosis confirmatory data limited our ability to do a correlation analysis of the clinical and radiological findings. Additionally, the study utilized the radiologist’s opinion as the ground truth, which may not always align perfectly with the final diagnosis.

## 6. Conclusions

Within the limitations specified above, this evaluation adds to the growing body of evidence that AI-based CAD software can serve as a potent adjunct to radiologists and clinicians in diagnostic workflows. The high diagnostic accuracy could lead to a reduction in workload and potentially address challenges associated with backlogs and diagnostic delays. Future prospective, real-world, multi-center studies could be the next step to validate the potential of AI in routine workflows. Additionally, such studies could facilitate the measurement of the tolerable limit of false-positive cases and the assessment of the potential risks of AI use in routine workflow settings.

## Figures and Tables

**Table 1:** Dataset characteristics. GP: general practice; A&E: Accident and Emergency; IP: inpatient; OP: outpatient; SD: standard deviation; FC: Fujifilm Corporation; and PMS: Philips Medical System.

Attribute	GP	A&E	IP	OP	Total
Scans Count	255 (25.6%)	246 (24.7%)	249 (25.0%)	243 (24.4%)	993 (100%)
**Demographic**					
Age (Mean)	65.50	66.55	62.67	65.48	65.05
Age (SD)	22.34	21.45	23.02	21.07	21.92
Female	121 (47.5%)	115 (46.7%)	123(49.4%)	135 (55.6%)	494 (49.7%)
Male	134 (52.5%)	131 (53.3%)	126 (50.6%)	108 (44.4%)	499 (50.3%)
**Manufacturer**					
FC	90 (35.3%)	97 (39.5%)	89 (35.7%)	91 (37.4%)	367 (36.9%)
PMS	144 (56.5%)	130 (52.8%)	134 (53.9%)	127 (52.3%)	535 (53.9%)
Kodak	21 (8.2%)	19 (7.7%)	26 (10.4%)	25 (10.3%)	91 (9.2%)
**Prevalence**					
Any abnormality	109	155	194	145	603
Nodule	2	6	6	7	21
Pneumothorax	1	4	10	1	16
Opacity	100	141	181	130	551
Pleural effusion	20	42	74	44	180
Hilar enlargement	4	11	9	10	34
Cardiomegaly	11	17	22	15	65

**Table 2:** Performance evaluation of DL-based algorithm at the optimal threshold for stratification of CXRs as normal or abnormal. SN: sensitivity; PPV: positive predictive value; SP: specificity; NPV: negative predictive value.

Target	SN (95% CI)	PPV (95% CI)	SP (95% CI)	NPV (95% CI)
Any abnormality	99.6 (98.7–99.9)	82.5 (79.6–85.1)	67.4 (62.3–71.8)	99.2 (97.2–99.7)
Nodule	85.7 (65.3–95.0)	11.8 (07.6–17.9)	86.2 (83.9–88.2)	99.6 (98.9–99.8)
Opacity	96.5 (94.6–97.7)	81.0 (77.8–83.8)	71.6 (67.2–75.6)	94.3 (91.3–96.3)
Pleural effusion	89.4 (84.1–93.1)	64.1 (58.0–69.8)	88.9 (86.5–90.9)	97.4 (96.0–98.3)
Hilar enlargement	79.4 (63.2–89.6)	21.6 (15.2–29.6)	89.7(87.7–91.5)	99.1 (98.3–99.6)
Pneumothorax	93.7 (71.6–98.8)	88.2 (65.6–96.7)	99.7 (99.2–99.9)	99.8 (99.4–99.9)
Cardiomegaly	63.0 (50.9–73.7)	36.8 (30.8–49.4)	93.3 (91.5–94.7)	97.3 (96.0–98.1)

**Table 3:** Imaging department-based sub-group performance for classifying CXR images as normal or abnormal scans using a DL-based algorithm at the optimum threshold. Dept: Department; SN: sensitivity; PPV: positive predictive value; SP: specificity; NPV: negative predictive value; GP: general practice; A&E: Accident and Emergency; IP: inpatient; OP: outpatient; Abnormal: CXRs classified as abnormal by at least one radiologist; Normal: CXRs classified as normal by both radiologists.

Department	Number of Images		SN (95% CI)	PPV (95% CI)	SP (95% CI)	NPV (95% CI)
	Normal	Abnormal				
GP	146	109	100 (96.5–100)	69.8 (62.2–76.5)	67.8 (59.8–74.8)	100 (96.2–100)
A&E	91	155	99.3 (96.4–99.8)	82.7 (76.7–87.5)	64.8 (54.6–73.8)	98.3 (91.1–99.7)
IP	55	194	99.4 (97.1–99.9)	89.7 (84.9–93.1)	61.0 (47.1–71.8)	97.0 (85.0–99.4)
OP	98	145	100 (97.4–100)	84.7 (78.6–89.4)	73.4 (63.9–81.2)	100 (94.9–100)

**Table 4:** Sub-group analysis for classifying CXR images as normal or abnormal scans using a DL-based algorithm at the optimum threshold. M: male; F: female; FC: Fujifilm Corporation; PMS: Philips Medical Systems; NI: number of images; SN: sensitivity; PPV: positive predictive value; SP: specificity; NPV: negative predictive value; Abnormal: CXRs classified as abnormal by at least one radiologist; Normal: CXRs classified as normal by both radiologists.

Group		Number of Images		SN (95% CI)	PPV (95% CI)	SP (95% CI)	NPV (95% CI)
		Normal	Abnormal				
Gender	M	195	304	100 (98.7–100)	81.5 (77.2–85.1)	64.6 (57.6–85.1)	100 (97.0–100)
	F	195	299	99.3 (97.5–99.8)	83.6 (79.4–87.1)	70.2 (63.4–76.2)	98.5 (94.9–99.6)
Age	18–52	107	147	100 (97.4–100)	80.3 (73.9–85.4)	66.3 (56.9–74.6)	100 (94.8–100)
	53–71	91	154	99.3 (96.4–99.8)	84.5 (78.5–89.0)	69.2 (59.1–77.7)	98.4 (91.6–99.7)
	≥72	192	301	99.6 (98.1–99.9)	82.6 (78.4–86.1)	67.1 (60.2–73.4)	99.2 (95.7–99.8)
Manufacturer	FC	144	223	99.1 (96.7–99.7)	85.0 (80.1–88.8)	72.9 (65.1–79.5)	98.1 (93.4–99.4)
	PMS	220	315	100 (98.7–100)	79.5 (75.2–83.2)	63.0 (56.4–69.1)	100 (97.2–100)
	Kodak	27	64	100 (94.3–100)	90.1 (81.0–95.1)	74.0 (55.3–86.8)	100 (83.8–100)

## Data Availability

Data are contained within the article.

## Appendix A

### Appendix A.1. Algorithm Description

Deep learning is a form of machine learning in which the hypothesis set is composed of neural networks (convolutional neural networks in our case). qXR uses deep learning models to detect abnormalities in chest X-rays. Training these models typically requires a large amount of labeled data. The development dataset for qXR contained more than 4 million chest X-rays with corresponding radiologist reports. Since these reports were in a free-text format, a custom natural language processing (NLP) algorithm was used to parse and extract labels that could be used for training. Although using these labels as the gold standard is not ideal, labeling the images in this manner enabled the use of the entire development dataset for training, with experts annotating only a fraction of the dataset.

In addition to identifying normal X-rays, the algorithms can detect individual chest X-ray findings like pleural effusions, cardiomegaly, parenchymal opacities, Hilar enlargements, nodules, masses, and devices.

### Appendix A.2. Natural Language Processing (NLP) Algorithm

The NLP algorithm was constructed using a thoracic imaging glossary, curated by a panel of radiologists and tailored to be consistent with the predefined abnormality definitions. This algorithm is rule-based as opposed to machine learning-based. The developers observed that rule-based systems performed better than learned methods, probably because of the vast amount of domain-specific medical knowledge that needed to be incorporated, which would have required large amounts of annotated data. The final NLP algorithm used to extract labels was essentially a large set of rules.

Since data were collected from multiple sources, the reporting standards were not consistent. The same finding could be noted in several different ways. For example, the following expressions were used to report the finding of a blunted costophrenic angle:

- CP angle is obliterated.
- Hazy costophrenic angles.
- Obscured CP angle.

All the words used to report the various findings were collected and rules were created for each finding. As an illustration, the following rule was used to capture the above three variations of a blunted CP angle:

((angle&(blunt|obscur|oblitera|haz|opaci))

The rule is considered positive if a sentence contains the words ‘angle’ and ‘blunted’ or their synonyms. In addition to such rules, there may be a hierarchical structure in the findings. For example, as per the definition used, opacity is considered positive if either edema, consolidation, ground glass, etc., are positive. Therefore, an ontology of findings and rules was created to address this hierarchy. As an illustration, the following rule captures this hierarchy for opacity based on the definition.

[opacity] rule = ((opacit & !( & collapse)) | infiltrate | hyperdensit) hierarchy = (edema | groundglass | consolidation | ... )

The top-level rule, ‘abnormal’, is an OR of all the individual findings. In addition to these rules for extracting mentions of abnormal findings from reports, negation detection, uncertainty detection, and a set of standard NLP techniques were employed to obtain the final labels, accounting for formatting and grammatical issues. Additionally, qualifiers like left, right, upper zone, etc., were extracted and served as additional labels for a given image.

### Appendix A.3. Abnormality Detection Algorithms

The AI algorithms used were trained to output classification labels and segmentation masks, which pinpoint the abnormal regions in the chest X-ray. In addition to a top-level algorithm that detects any abnormal findings on a chest X-ray, there are separate detection pipelines that identify and localize the type of abnormality. The algorithm uses UNet++ for the segmentation task with EfficientNetv2 as the backbone. EfficientNetV2 represents a family of CNN architectures used to efficiently scale a model’s depth, width, and resolution, thereby optimizing the number of model parameters. UNet++ is a derivative of the UNet architecture that uses an encoder–decoder architecture with skip connections. The segmentation task was trained with pixel-level annotations performed by a team of radiologists, whereas the classification task was trained using NLP-generated labels. The final algorithms used NLP-derived labels from 4.4 million chest X-ray reports and expert-labeled free-hand annotations on about 1.6 million X-rays. Chest X-rays with abnormal findings according to the NLP algorithm were given priority for expert labeling. A set of 500,000 randomly sampled chest X-rays was set aside for internal testing. To boost the reliability of internal testing, each image in the internal test set was annotated by seven radiologists. For the classification task, a cross-entropy loss was used, and for the pixel-level segmentation task, a DICE-BCE loss, which combines the dice coefficient loss and binary cross-entropy loss, was used. A vanilla stochastic gradient descent optimizer with momentum was used for tuning the model parameters to optimize a weighted sum of the losses from the classification and segmentation tasks. All experiments were conducted on servers with eight V100 GPUs. The final algorithms were trained for about 150 epochs using a batch size of 8192.

### Appendix A.4. Selection of Operating Points

The outputs from the deep learning-based models were continuous variables. Two default thresholds were derived by following the procedure detailed below. The high-sensitivity operating points were used as the thresholds for this evaluation.

High sensitivity point: Select the operating point where the sensitivity is closest to 0.95. If the specificity >0.70 at this operating point, use this operating point. Otherwise, use an operating point where the sensitivity is just above 0.90 if available. If not, select the one closest to 0.90.

High specificity point: Select the operating point where the specificity is closest to 0.95. If the sensitivity > 0.70 at this operating point, use this operating point. Otherwise, use an operating point where the specificity is just above 0.90 if available. If not, select the one closest to 0.90.
